# Why Single-Cell Sequencing Has Promise in MDS

**DOI:** 10.3389/fonc.2021.769753

**Published:** 2021-12-02

**Authors:** Xuan Zhang, H. Leighton Grimes

**Affiliations:** ^1^ Division of Immunobiology and Center for Systems Immunology, Cincinnati Children’s Hospital Medical Center, Cincinnati, OH, United States; ^2^ Division of Experimental Hematology and Cancer Biology, Cincinnati Children's Hospital Medical Center, Cincinnati, OH, United States; ^3^ Department of Pediatrics, University of Cincinnati, Cincinnati, OH, United States

**Keywords:** myelodysplastic syndrome (MDS), single-cell sequencing (SCS), single cell multi-omics profiling, hematopoiesis, myeloid malignancies

## Abstract

Myelodysplastic syndromes (MDS) are a heterogeneous group of diseases characterized by ineffective hematopoiesis. The risk of MDS is associated with aging and the accumulation of somatic mutations in hematopoietic stem cells and progenitors (HSPC). While advances in DNA sequencing in the past decade unveiled clonal selection driven by mutations in MDS, it is unclear at which stage the HSPCs are trapped or what prevents mature cells output. Single-cell-sequencing techniques in recent years have revolutionized our understanding of normal hematopoiesis by identifying the transitional cell states between classical hematopoietic hierarchy stages, and most importantly the biological activities behind cell differentiation and lineage commitment. Emerging studies have adapted these powerful tools to investigate normal hematopoiesis as well as the clonal heterogeneity in myeloid malignancies and provide a progressive description of disease pathogenesis. This review summarizes the potential of growing single-cell-sequencing techniques, the evolving efforts to elucidate hematopoiesis in physiological conditions and MDS at single-cell resolution, and discuss how they may fill the gaps in our current understanding of MDS biology.

## Introduction

Hematopoietic stem cells give rise to a variety of mature cells in a programmed way as determined by their epigenetic, transcriptomic, and spatial information (1, 2). Recurrent intrinsic alterations including somatic mutations and extrinsic influences such as inflammation are found to contribute to imbalanced hematopoiesis (3, 4). Clinical presentations vary from ineffective erythropoiesis in MDS to uncontrolled clonal proliferation as observed in myeloid proliferative neoplasm (MPN) and acute myeloid leukemia (AML). It has been well established that MDS is a stem cell disease characterized by a collection of cytogenetically abnormal cells predominately in the stem and progenitor compartments (5); Researchers and physicians often struggle with three central questions when they try to understand MDS. First, how does the malignant clonal architecture look like in MDS? Second, what caused this architecture and the dynamic of this structure following therapeutic intervention. Third, why the cytogenetically normal cells cannot maintain normal hematopoiesis. These questions involve multiple aspects of hematopoiesis and stem cell biology and have been difficult to answer due to technical difficulties to accurately characterize these cells and further define the determining cellular activities of their fates. Single-cell sequencing studies have reinvented how we interpret normal hematopoiesis, and with emerging multiomic techniques unveiled the crucial regulatory activities in HSPC lineage priming and commitment. Now it is time to use these delicate approaches to identify and characterize the malignant HSPCs in MDS, which shall provide not only insights into MDS pathogenesis but also novel rationales for clinical studies.

## Single-Cell Profiling, an Era of Multiomics

The very basis to answer the three essential questions mandates an accurate description of the cell types and states, furthermore the regulatory network that places them in various stages of hematopoiesis. Thanks to flow cytometry, functional colony assays, and transplantation/adoptive transfer, the hematopoietic system is one of the best characterized for stem cell populations and differentiation hierarchy. Historically, hematopoiesis has been represented by a hierarchy of flow cytometry-defined populations e.g. granulocyte monocyte progenitor (GMP), common myeloid progenitor (CMP). Work by Pronk et al, delineated functional and surface-marker heterogeneity within CMP/GMP gate populations (6). However, while recent single-cell sequencing studies in murine models are redefining the murine hematopoietic differentiation tree, most researchers realized that CMP and GMP were only flow gates, and not homogeneous progenitor populations, only after single-cell RNA sequencing (scRNA-seq) unveiled dramatic heterogeneity within, and transitional states between, functionally- and phenotypically-defined cell states. Currently, cytometry-defined gates are understood to constitute marrow cell fractions that are enriched for stem and/or progenitors. Of note, there could be a disconnect between the transcriptionally defined cell states by scRNA-seq and the function/capability of these cells since proteins exert to the eventual cellular process; mRNA and protein levels do not correlate well as there are additional post-transcriptional regulations in play (7). On top of that, human hematopoietic stem cells (HSCs) and multi-potent progenitors (MPP) transcriptomes are not easily resolved by informatic clustering methods. Multi-omic technologies that provide additional proteomic and/or epigenomic information are expected to reveal functionally relevant HSC and MPP clusters and underlying coherent gene regulatory networks as a necessary precursor to facilitate understanding normal HSPCs and those changes induced in MDS.

Ground-breaking single-cell sequencing tools are being developed every year. In contrast to early days well-based single-cell sequencing that can only profile a few hundreds of cells, single-cell partition methods nowadays could expand the number of captured cells close to a million eg. 10X Genomics High-throughput with indexing. Technologic innovations are trailed by informatics methods to deal with the growing data size and types. Currently, the single-cell field is focused on the integration of multiple data features to resolve important questions concerning potential disconnects between single-cell data and mechanistic insights. Notably, because single-cell technologies capture only a modest portion of cellular RNA, and gene transcription occurs in bursts, scRNA-seq data are sparse. From this technically imposed limitation reinforced by biases present in many informatics pipelines (e.g. dimensional reduction), researchers have concluded that the continuous nature of sparse transcriptome data suggests that hematopoiesis is itself a continuum from primitive HSC to mature cells in the peripheral blood (8–12). This is a surprising hypothesis in that a hematopoietic continuum would require a lack of stability in all cell states from HSC to differentiated progeny. Note that combined scRNA-seq and lineage-tracing showed that gradual changes in gene expression may not always relate to a change in functional output (13). Thus, within the continuum of data, functionally relevant cell states may exist. Our work (and that of many others) suggests that differentiation is likely represented by transition through more and less stable cell states that are controlled by the coordinated action of sets of transcription factors (14, 15). Here, it is important to focus analyses on differentiation-associated gene expression programs, as opposed to other cellular programs simultaneously running within cells (e.g. cell cycle, circadian rhythm, etc.) which together constitute a cell state. ICGS2 and URD are excellent methods to isolate developmentally relevant gene expression programs (16, 17). However, human HSC and MPP are notoriously difficult to cluster and some researchers have posited that differences between such primitive populations are a “cloud-like” continuum. It is likely that the integration of single-cell multi-omics data will be required to clearly identify both normal HSPC subsets, and then by comparison those affected by MDS.

Similar to the discordance between RNA and protein levels, epigenetic marks (eg. histone modifications, transcription factors) could favor a specific lineage priming without/not yet influencing RNA expression, thereby further segregating HSPCs by fate anticipation and adding complexity to the cell state. ATAC-seq (Assay for Transposase-Accessible Chromatin using sequencing; pronounced “attack”, not “ay-tack”) utilizes an adaptor-loaded transposase to mark accessible chromatin regions, a concept similar to other broad chromatin accessibility assays like DNase-Seq or FAIRE-Seq. The data reveals transposon-accessible chromatin, which is inferred to be open chromatin. However, ATAC-seq requires fewer cells and quickly became compatible with single-cell capture platforms to infer the transposon-accessible landscape in each cell (18). Based on the inferred transcription factor binding sequences within open chromatin it is possible to reconstruct the hematopoietic architecture of progenitors (but not HSC/MPP) using scATAC-seq data (1). Combined with scRNA-seq data from well-defined phenotypic cell populations (1, 19), the transcriptome can be integrated with the scATAC profile. Popular algorithms like Cicero connect regulatory elements to their putative target gene promoters (20). However, there are two limitations of this approach. Firstly, the effect of these accessible regions discovered by TN5 relies heavily on scanning the binding motifs and their related transcriptional factors. The subsequent gene expression may display a discordance with the state of chromatin accessibility, especially in stem and progenitor cells where the chromatin is open(primed) for lineage decisive gene expression, yet the genes are not yet expressed. Therefore, simultaneous capture of both chromatin accessible information and transcriptome should provide a more accurate description of MDS HSPCs than integrating scRNA-seq and scATAC data. Secondly, linkages between promoters and putative enhancers are only inferred. To gain validation for inferred promoter-enhancer interaction in small cell numbers, high-throughput chromatin conformation assays such as promoter capture Hi-C (PCHiC)/single-cell Hi-C (21), PLAC-seq/HiCHIP with CTCF and RNApolII can connect proximal and distal regulatory elements to dynamic gene expression (22, 23). These methods could also help understand the interplay of co-mutations in cohesin components that are frequently mutated in MDS and AML eg. STAG2 with SRSF2, ASXL1, and RUNX1 (24). The underlying chromatin permissiveness alteration could be explicated by the histone modifications such as H3K27ac(enhancer), H3K4me3(promoter) and H3K27me3(repressor) or transcriptional factors. Recently, CUT&Tag, a technology similar but superior to CHIP-seq in terms of signal to noise that uses antibodies to direct TN5 mediated sequence insertion around desired histone modifications was adopted to single-cell level (25–27). Notably, without using conA beads in sample preparation for 10X genomic platform, the vast majority of the nuclei are lost prior to chip loading which makes it questionable whether the remaining nuclei are still representative of the original population. On the other hand, well-based single cell partition methods are compatible with conA beads and could be viable.

Novel multi-omic technologies with the help of higher throughput platforms like the gel bead-in emulsion (GEM) are emerging to simultaneously reveal multiple features from single cells. Simultaneous detection of RNA and surface proteins (CITE-seq and REAP-seq) using antibody-derived tags(ADT) exploits DNA oligos to quantify antigens and can be captured by poly(dT)(TotalSeq-A) or partial Read1(TotalSeq-B) at 3’ or by template switch oligo(TotalSeq-C) at 5’ (28, 29). A caution here is that commercially available ADT-conjugated antibody mixes are not necessarily titrated for hematopoietic cells, and definitely not on rare human CD34+ cells. Newer commercial multiome platforms by 10X Genomics provide a much more convenient approach compared to labor-intensive well-based SHARE-seq (30) by using gel beads that contain capture sequences for both RNA by 3’ polyA tail and transposed DNA therefore allowing simultaneous capture of ATAC and nuclear RNA. Moreover, several new methods attempt to link surface proteins and/or RNA to transposon-accessible chromatin. First, ATAC with select antigen profiling by sequencing (ASAP-seq) explored fixation to retain surface-bound ADT during nuclei permeabilized for TN5 transposition but lacks RNA capture (31). Second, fixation-free single-cell tri-modal information capture of Transcriptome, Epitopes and ATAC (TEA-seq) was established by Swanson et al (32). Chen et al. described single-cell Nuclear protein Epitope, chromatin Accessibility, and Transcriptome sequencing (NEAT-seq) which measures the molecular consequences of continuous changes in transcriptional factor levels. These simultaneous captures of different layers of information avoid the step of identifying common patterns between each layer for integration (33) and thus will provide an unparalleled accuracy of profiling individual hematopoietic cells and infer the clonal hierarchy. Note that informatics methods to exploit the strengths of each feature to cluster these data sets are still in early stages. Functional validation and readout are required (in addition to the single-cell data) to consolidate the claims.

While single-cell techniques and informatics methods are capturing layers of information from a cell exhaustively, the focus has extended beyond what is in a cell, to spatial information. It is well recognized that hematopoiesis happens in a specific niche. Recent scRNA-seq studies have resolved bone-marrow cellular heterogeneity, identified the pro-hematopoietic factors, and characterized their secreting cells (34–36). Zhang et al. showed that murine HSPCs undergo lineage commitment and differentiation in a spatially restricted way (37, 38). Specifically, they showed that monocytic production was spatially separated from other lineages and that spatially-controlled monocytic production was influenced by a small subpopulation of endothelial cells producing the monopoietic cytokine Csf1. Thus, distinct blood vessel populations produce local cues which instruct spatial hematopoietic organization (38). Baccin et al. combined scRNA-seq and RNA sequencing of laser microdissected single cells to identify two subpopulations of murine Cxcl12-abundant reticular (CAR) cell subsets, of which Adipo-CAR cells differentially localize to sinusoidal or arteriolar surfaces, acting as professional cytokine secreting cells to establish distinct peri-vascular micro-niches to support HSPCs (39). Given the established pro-inflammatory niche in MDS and defects in the stromal (40), it is intriguing to map MDS cells in murine models(not possible from MDS patients) to the stromal cells in the niche to first see if MDS cells utilize a different spatial organization that disapproves erythropoiesis, second their physical interaction with immune cells which may help the development of engineered cells products.

## The Biology of MDS, Imbalanced Hematopoiesis

Myelodysplastic syndromes (MDS) are a group of hematologic disorders characterized by ineffective hematopoiesis and cytopenias due to blockade in differentiation or removal of more mature progenitors *via* apoptosis (41). The median age at diagnosis is 70 years and MDS is on the rise with aging of the population and in cancer survivors (treatment-related MDS; tMDS). The clinical presentation of MDS is variable, such as the type and severity of cytopenia, bone-marrow dysplasia, and cytogenetic abnormalities. Survival also varies greatly by the subtype of MDS, according to the Revised International Prognostic Scoring System (IPSS-R) which stratifies patients by the severity of anemia and cytopenia, blast count, and cytogenetics (42). The risk of transformation to AML ranges from a few months in patients with higher-risk MDS(HR-MDS) and tMDS, to several years in those with lower-risk MDS(LR-MDS) (42).

Previous studies characterized the HSPCs from MDS patients *via* surface markers and observed stage-specific phenotypic alterations which have been reviewed in detail by Shastri et al (43). Notably, while symptoms such as anemia and thrombocytopenia can be attributed to the decrease of Lin−CD34+CD38+CD123−CD45RA− megakaryocyte erythroid progenitor (MEP), the phenotypic HSC are expanded in all types of MDS. Furthermore, expansion of Lin−CD34+CD38+CD123+CD45RA− CMP is observed in LR-MDS, whereas HR-MDS is associated with expansion of both Lin−CD34+CD38−CD90+ long-term HSC(LT-HSC) (41, 44, 45) and Lin−CD34+CD38+CD123+CD45RA+ GMP(similar to findings from murine AML models in which GMP contain an LSC) (46, 47). The reduction of GMP in LR-MDS is believed due to increased apoptosis and phagocytosis, which is reverted in HR-MDS through upregulation of CD47, a “don’t eat me” signal (41). These phenotypic populations can display additional surface markers which differentially mark MDS HSPCs for a higher risk such as CD47, interleukin-1-receptor accessory protein (IL1RAP), CD99, CD123, and TIM3 (5). In any case, the expanded phenotypically-defined progenitor cells cannot efficiently differentiate to mature cells that exit bone marrow, and if expansion reaches an uncontrolled leukemic state, may compete with normal HSPCs and damage the niche further thwarting normal hematopoiesis (40).

The efforts to describe the clonal architecture of MDS however cannot reveal the underlying cellular process in those cells, and the cell populations based on what we have learned from scRNA-seq data from normal hematopoiesis, are heterogeneous. Unfortunately, a comprehensive assessment of cells from human MDS with aged match reference is lacking so far. Interestingly, Zhu et al. utilized well-based single-cell RNA sequencing to examine the full-length transcriptome of HSPCs in 15 treatment-naïve patients with aplastic anemia (AA), a disease which frequently transforms to MDS. They observed relative enrichment of neutrophil progenitors, but marked depletion of HSCs/MPPs, MEPs, and monocyte-dendritic progenitors (MDP). In addition, many spliceosome genes were differentially expressed in the remaining HSPCs that possibly contribute to the 50.7% of altered splicing events shared between AA and MDS (48). Their findings are in line with the high frequency of splicing factor mutations in MDS such as SF3B1 and SRSF2 which impairs erythroid cell maturation (49) or skew megakaryo‐erythroid differentiation toward megakaryocytes respectively (50), suggesting the possible role of alternative splicing in erythroid lineage commitment. Highly multiplexed mass flow cytometry that exploits both surface markers and intracellular signaling changes such as phosphorylation could also be helpful to address this question. Behbehani et al. took advantage of CyTOF mass cytometry to examine 34 parameters in 23 MDS and 3 ICUS patients’ bone marrow samples. While the frequency of committed progenitors and fully differentiated cells in the monocyte and granulocyte lineages were relatively reduced in low-risk MDS, higher-risk MDS samples exhibited abnormal immunophenotypic myeloid cells that resemble myeloid-derived-suppressor cells characterized by moderate expression of CD64, CD11b, CD44, CD45, CD45RA and lack of HLA-DR, CD14 and CD15 (51).

## The Pathogenesis of MDS and Its Genetics

The pathogenesis of MDS is not fully understood, yet advances in genomic sequencing have established the cytogenetic and mutational spectrum of MDS, suggesting a similar “multiple-hit” model that has been applied to many other cancers. Namely, hematopoietic stem and progenitor cells (HSPCs) are blocked in differentiation due to the accumulation of somatic gene mutations in key cellular processes.

To date, more than 30 driver genes have been identified in MDS. Among those, the most frequently mutated genes affect DNA methylation (*DNMT3A, TET2, IDH1, IDH2, and WT1*), chromatin modification (*EZH2, ASXL1*, and *KMT2*), RNA splicing (*SF3B1, SRSF2, U2AF1*, and *SF1*), and transcription factors (*TP53, RUNX1*) *(* 4, 52, 53). These mutations are not unique to MDS. Common MDS-driver mutations (especially *TET2* and *DNMT3A*) can be found in elderly individuals without any clinical symptoms. This non-malignant type of clonal outgrowth of hematopoietic cells is referred to as clonal hematopoiesis of indeterminate potential (CHIP) (54, 55). In addition, primary and secondary AML share the same pool of mutational targets, yet the frequencies of mutations in FLT3, PTPEN11, NRAS, NPM1, IDH1, and IDH2 are more prevalent in AML compared to MDS (4, 56). Notably, the variable combinations of mutations make it difficult to predict prognosis or response to front-line therapeutic agents; albeit HR-MDS tend to present higher numbers of mutations compared to LR-MDS (57–59). There are notable exceptions. While *TP53* mutation is associated with a complex karyotype (especially del(5q) and 17qLOH), and generally predicts a dismal outcome regardless of therapeutic interventions (60, 61), *SF3B1* mutation (which is commonly found in MDS ring sideroblast; MDS-RS) is associated with favorable outcome and is now recognized as a discrete type of myeloid neoplasm (62).

It is intriguing that despite the presence of tumor-driver mutations and 11 to 13-fold increased risk to malignant transformation, only 0.5-1% convert to malignancies every year (63–65). The additional mutation “hits” required for transformation and their sequential acquisition remain elusive; Palomo et al. performed a large MDS/MPN cohort sequencing study which revealed specific gene-mutation combinations, and their order of acquisition. For example, *SRSF2* and *ASXL1* mutations (but not *RUNX1* mutation) were preceded by *TET2* mutation (66). However, it is difficult to interpret whether two or more detected mutations are in the same cell or are in different subclones, especially in LR-MDS where mutation variant allele frequency (VAF) can be low. Clues have recently come from single-cell analysis. Recent advances in single-cell DNA sequencing techniques such as single-cell index-sorting PCR or microfluid-based platforms have enabled researchers to build mutational clonal architectures of MDS. HSPCs isolated from del(5q) MDS patient bone marrow were captured for single-cell copy number analysis and genotyping. High intertumoral heterogeneity was highlighted as compared to bulk sequencing (67). Silva-Coelh et al. used whole-exome and targeted deep sequencing at multiple time points to explore clonal evolution in MDS patients receiving supportive care, or lenalidomide. They performed sequencing on single-cell-derived clones to confirm the coexistence of ancestral mutations. Both linear and branched mutational patterns of clonal evolution were identified (68). However, single-cell DNA sequencing alone only tells whether a cell is cytogenetically normal or not, the identity of that cell is missing.

With the help of flow cytometry, based on the expression of known leukemic stem cell (LSC) markers, Chen et al. sorted both pre-malignant and malignant stem cells from matched MDS and MDS-AML samples, then performed single-cell targeted sequencing. They demonstrated a non-linear model, where initiating-mutation-bearing cells can either develop into MDS or AML based on acquired mutations. MDS studies are limited due to the scarcity of sample material (e.g. hypocellular marrow). In contrast, single-cell genomic sequencing studies on MPN and AML demonstrated how mutations collaboratively contribute to clonal dominance or promote clonal expansion. Interestingly, although signaling pathway mutations such as *PTPN11* and *FLT3* are acquired by AML clones during transformation, they rarely coexist in the same cell; suggesting overlapping oncogenic functions (69, 70). Recently, Biolegend has expanded their Totalseq antibodies(D) to work with Mission Bio’s single-cell DNA sequencing platform Tapestri, unlocking a simultaneous discovery of frequent mutations as well as surface markers that define cell types. The addition of surface markers may serve as anchors to integrate DNA mutation information with CITE-seq data. It could still be challenging because of the limited antibody panel (<50) and very low recovery rate of Tapestri platform. Alternatively, genotyping from cDNA can be achieved with some limitations such as the position of the mutation to 3’ or 5’, and the mutation must be verified by bulk DNA sequencing first. Given the rapid advances in single-cell technologies, it is promising that soon we could obtain both mutation information and identity of a single cell, thereby distinguishing the cytogenetically abnormal cells and their clonal path in MDS.

## Therapies That Restore Hematopoiesis

MDS is a very heterogeneous disease and the majority of MDS patients do not progress to AML. For example, IPSS-R LR-MDS patients take 9.4 years on average for ~25% of patients to progress to AML (42). Thus, the therapy for HR-MDS and LR-MDS are different. However, hypomethylating agents (HMA) such as decitabine and azacytidine are commonly found on regimens for both risk groups and can induce significant responses. Targeted inhibitors that were originally aimed at eliminating leukemic blast and LSC in AML have been frequently used in combination with HMA clinically to delay AML progression in HR-MDS. Most notably, the combination of Bcl2-inhibitor venetoclax plus the HMA azacitidine has recently been granted FDA-breakthrough therapy designation for treating naïve HR-MDS. Other inhibitors that target key driver mutations are being actively evaluated in phase II/III clinical trials include FLT3 inhibitor Quizartinib(NCT04493138), a putative mutant-p53 inhibitor APR-246/APR-548(NCT04638309), RARα Agonist SY-1425(NCT04797780), therapeutic antibodies either directly targeting malignant cells by antibody-dependent toxicity such as daratumumab(CD38) and talacotuzumab(CD123), or by modulating immune response such as pembrolizumab(PD-1), sabatolimab(TIM3) and magrolimab(CD47). On the other hand, contrary to cytotoxic methods targeting the uncontrolled proliferation of blasts, the management of LR-MDS aims at restoring hematopoiesis. HMA is frequently used in LR-MDS patients with neutropenia and/or thrombocytopenia, or in those who have failed (or are predicted to fail) other agents such as growth factors and lenalidomide (71). Other therapeutic approaches also display clinical efficacy in restoring erythropoiesis in MDS patients. For example, the Hypoxia-inducible factor (HIF) inhibitor roxadustat(NCT03263091) has entered a phase III trial focusing on improving anemia (72).

The TGF-β-pathway inhibitor luspatercept promotes erythroid maturation by decreasing the constitutively active SMAD2/SMAD3 signaling in MDS HSPCs. Luspatercept is especially effective in MDS-RS or *SF3B1*-mutated MDS [77% vs 40% in non-mutated (73)] possibly by alleviating the impact from proinflammatory microenvironment (74). In the MEDALIST trial, a total of 180 patients diagnosed with MDS-RS achieved 59% erythroid response during 1-48 weeks and 38% achieved transfusion independence at >8 weeks (75). In comparison, Lenalidomide restores erythropoiesis in 5q del MDS by synthetic lethality from the degradation of haplodeficient proteins encoded within the 5q deleted region (76).

In contrast to the specific mode of action of luspatercept and lenalidomide in MDS-RS and 5q del MDS respectively, the mechanism behind low-dose HMA-induced hematopoiesis restoration in MDS is not fully understood. Understanding which genes need to be demethylated is complex because aging dramatically affects the hematopoietic methylome landscape, and there are lineage-specific methylome changes. Indeed, DNA methylation profiles can predict cell type throughout human hematopoietic lineages (77). Adelman et al. found that aging is associated with reprogramming DNA methylation along with a reduction of activation-associated histone modifications (e.g. H3K27ac, H3K4me1, and H3K4me3). Many of these changes were coincident with those found in human AML, revealing that most of the plethora of methylome changes seen when comparing older HSC, MDS, or AML to cord blood cells is likely indicative of aging and not transformation (78).

Despite the strong rationale of using HMA to overcome blocked hematopoiesis in MDS; one-third of patients achieve overall objective response and one-half maintain stable disease after HMA treatment. Unfortunately, the response duration is short (7 months) and the prognosis is poor even in LR-MDS. In a large cohort study, the median transformation-free survival (TFS) and overall survival (OS) were 15 months and 17 months respectively (79). HMA-induced resolution of anemia is associated with reduction of blast and an overall number of cytogenetically abnormal cells in bone marrow as well as repopulation of cytogenetically normal LT-HSCs, yet the cytogenetically abnormal LT-HSCs persist and contribute to transformation to AML (44, 80). These findings suggest that HMA treatment cannot eradicate MDS HSPCs. Given that the cytogenetically abnormal HSC in MDS can reconstitute a limited spectrum of clonal architecture such as the re-expansion of progenitor compartments, it reminds us that MDS is a stem cell disease. When HMA and other agents restore hematopoiesis, which blockade do they lift, and why cytogenetically abnormal HSC are not affected remains elusive.

Clues to how HMA treatment works (when successful) come from Kanagal-Shamanna et al, who found that HMA treatment of MDS-RS HSPCs predominantly affects erythroid differentiation using scRNA-seq. Genes involved in translation, oxidative phosphorylation, and cell cycle progression were found differentially expressed between MDS-RS HSPCs and those in healthy donors. In patients with MDS-RS, erythroid output was arrested at the orthochromatic normoblasts (ON) to pre-reticulocyte transition. Interestingly, comparing paired pre-and post-HMA treatment samples, aberrant differentiation of MDS-RS HSPCs towards the erythroid lineage is persistent, but HMA temporarily induced the MDS-RS ONs into reticulocytes and facilitated their release into peripheral blood (81). It would be interesting to examine the HSPCs by single-cell profiling from patients with other types of MDS that achieved long-term remission vs those that failed HMA. These findings may help to develop approaches to achieve lasting remission in patients with MDS.

## MDS HPSCs and Their Normal Counterparts

Knowing the characteristics of normal cells should facilitate understanding their malignant counterparts, and thus provide answers to why HSCs in MDS are resistant to current front-line therapies such as HMA. Ganan-Gomez et al. showed heterogeneous HSC architecture in cells from two MDS patients’ bone marrow, which converge at later myeloid progenitor states (82). Wu et al. looked at the single-cell transcriptome of bone marrow cells from 8 patients with pathogenic GATA2 mutations and myelodysplasia. Deficiency in lymphoid/myeloid progenitors was observed and stem cells in patients had dysregulated gene expression implying GATA2 mutation impacts hematopoiesis prior to lineage commitment (83). Izzo et al. analyzed the loss of the DNA methylation genes *DNMT3A* and *TET2* (which are among the most frequently mutated founding genes in MDS) through scRNA-seq, chromatin accessibility, and methylome; showing *TET2* KO hypermethylation led to myelomonocytic skews in HSC priming. In contrast, Dnmt3a KO-induced hypomethylation differentially affected DNA-binding motifs between myelomonocytic and erythroid-associated transcription factors to skew erythropoiesis (84). By analogy, these mouse-model findings suggest that in very early MDS (or during CHIP) myeloid lineage priming in MDS HSCs might already be deregulated.

Examples of where integrated single-cell technology could attack MDS come from its use in other hematopoietic diseases (where the material is not as limiting). Single-cell genotyping of transcriptomes (GoT) from 10X genomic cDNA libraries allows targeted detection of gene mutations in each cell, which further helps to compare the transcriptome between malignant versus normal cells in each cluster. Notably, the detection coverage is low, suffering from the general shorter transcripts in single-cell platforms (85). Van Galen et al. exploited AML intra-tumor heterogeneity by scRNA-seq and single-cell genotyping from cDNA. They particularly focused on FLT3 genotypes in malignant cell clusters; FLT3-ITD subclones were contained within primitive cells whereas FLT3-TKD subclones in the same tumor primarily contained differentiated cells. In addition, differentiated monocyte-like AML cells were found to potently inhibit T-cell activation *in vitro (* 86). Another group of researchers used similar microwell-based scRNA-seq, identified an intermediate state between HSC to differentiated populations which they recognized as the progenitor population in *de novo* AMLs (87). scATAC-seq also unveiled regulatory heterogeneity in AML cells; the clonally-derived LSCs might represent a clonal outgrowth of a rare cell type and/or intermediate differentiation state. This population was posited to have coopted regulatory programs and exist as stable intermediate cell states that are not normally observed in normal hematopoiesis (88). In an integrative approach using CITE-seq and scATAC-seq by Granja et al (89), leukemic cells from mixed-phenotype acute leukemias (MPAL) were projected to a subspace constructed based transcriptome and ATAC data from healthy individuals. The nearest normal cell, regardless of its cell-type boundaries, was used to compare the differentials. From the putative peak-to-gene linkages and transcription factors that regulate leukemia-specific genes, *RUNX1* was inferred to regulate CD69 and act as a potential oncogene in MPAL related to poor survival. Psaila et al. studied myelofibrosis with scRNA-seq and CyTOF and delineated a bias toward megakaryocyte differentiation among early multipotent stem/progenitor cells. By comparing disease-specific megakaryocyte progenitors to their normal counterparts, and validating with surface protein expression, G6B was identified as a potential immunotherapy target in myelofibrosis (90).

## Conclusion

**Figure 1:**
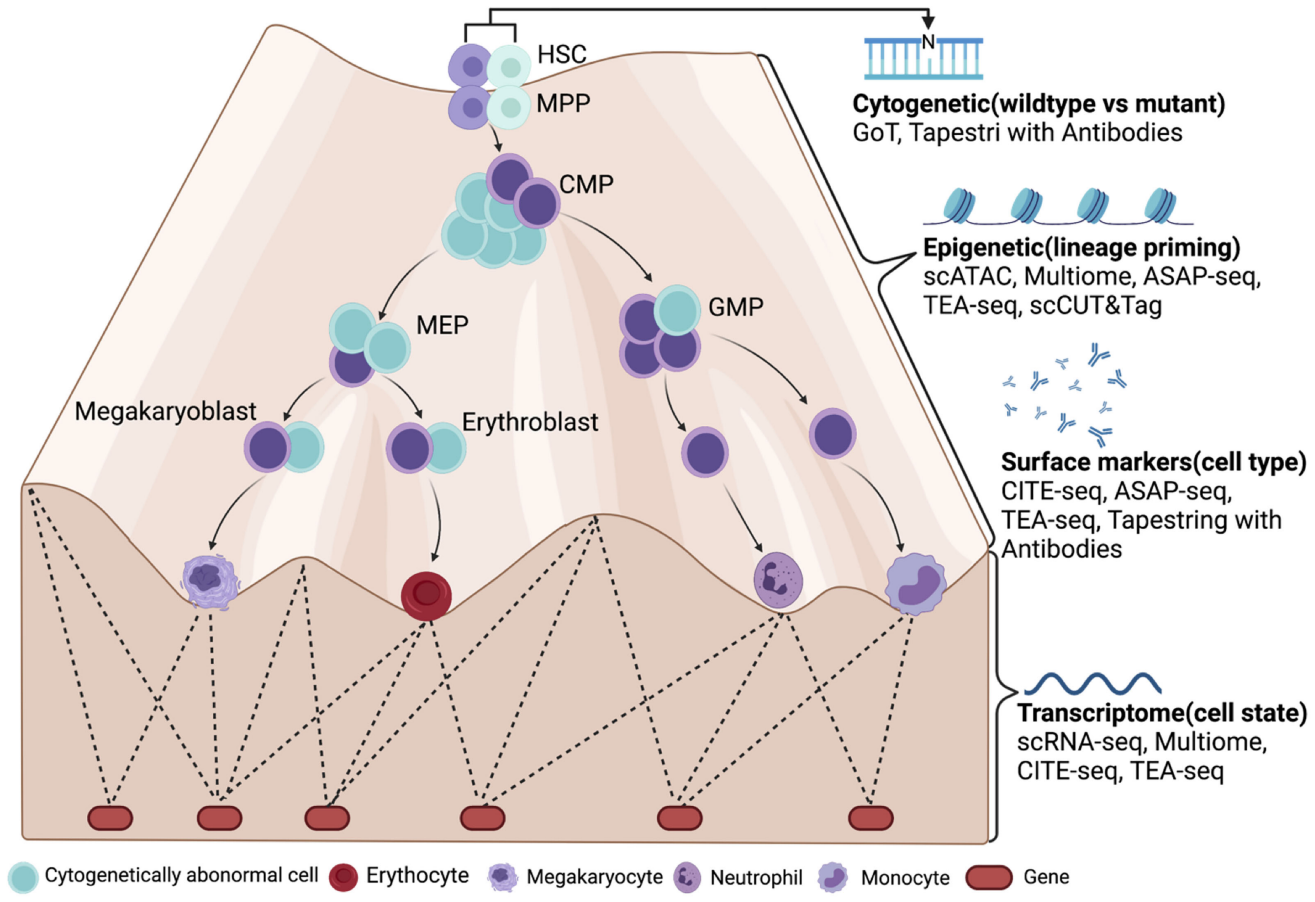
A future look of MDS at single-cell resolution with multiomic tools (LR-MDS as an example). Cytogenetically abnormal cells can be distinguished from normal cells by single-cell mutation profiling (cDNA or DNA) in MDS. Their differentiation stage and state are then defined by their surface marker expression and transcriptome. With the help from chromatin accessibility assays such as scATAC-seq, the epigenetic landscape is revealed by transcriptome and gene regulatory networks, which determines the most subtle change in lineage priming process. Figure was created with BioRender.com.

Our knowledge of the biology of MDS has been limited due to the availability of malignant cells and their complex interactions with normal hematopoiesis. The constant clonal evolution and relatively slow disease progression has been a historical challenge to design mouse models that can accurately model MDS. It is important to remember MDS unlike AML, represents a progression of clonal hematopoiesis where dominant cytogenetically abnormal HSPCs have not yet gained the pass to uncontrolled proliferation, the imbalanced output contributes to abnormal cells spanning multiple differentiation stages that result in the clinical phenotypes. Understanding the biology of MDS and how to treat it requires an accurate characterization of those cells in MDS; in which cell state they are at and regulatory networks that decide their fate. Emerging single-cell profiling techniques enable the precise characterization of individual cells and have already transformed our understanding of normal and malignant hematopoiesis. Recent studies have showcased the capacity of single-cell sequencing to discriminate between the heterogeneous subclones of cells in AML (as well as in MDS) by comparing their transcriptome and epigenome to the nearest normal cell/group. Although the number of single-cell studies of MDS is very limited at this moment, with the growing availability of multiomic single-cell techniques (**Figure 1**), we expect to see a definitive description of the malignant and normal hematopoiesis architecture in MDS as well as their fate determinant, which may provide actionable targets such as surface proteins that can be targeted by antibody or ligands or key signaling pathway that can be targeted by small-molecule inhibitors.

## Author Contributions

All authors: writing—original draft preparation. All authors contributed to the article and approved the submitted version.

## Funding

Partial support for this review came from R01CA253981, R21HL150678 and RC2DK122376 (to HLG).

## Conflict of Interest

The authors declare that the research was conducted in the absence of any commercial or financial relationships that could be construed as a potential conflict of interest.

## Publisher’s Note

All claims expressed in this article are solely those of the authors and do not necessarily represent those of their affiliated organizations, or those of the publisher, the editors and the reviewers. Any product that may be evaluated in this article, or claim that may be made by its manufacturer, is not guaranteed or endorsed by the publisher.
